# Fabricating electrospun cellulose nanofibre adsorbents for ion-exchange chromatography

**DOI:** 10.1016/j.chroma.2014.12.010

**Published:** 2015-01-09

**Authors:** Stewart R. Dods, Oliver Hardick, Bob Stevens, Daniel G. Bracewell

**Affiliations:** a Department of Biochemical Engineering, University College London, Bernard Katz Building, London WC1H 0AH, UK; b Innovations Technology Access Centre – Micro and Nanotechnology, Rutherford Appleton Laboratory, Science and Technology Facilities Council, Harwell Oxford, Didcot OX11 0QX, UK; c School of Science and Technology, Nottingham Trent University, Nottingham, NG1 4BU, UK

**Keywords:** Electrospinning, Convective mass transfer, Diethylaminoethyl, Carboxylate, TEMPO-mediated oxidation

## Abstract

Protein separation is an integral step in biopharmaceutical manufacture with diffusion-limited packed bed chromatography remaining the default choice for industry. Rapid bind-elute separation using convective mass transfer media offers advantages in productivity by operating at high flowrates. Electrospun nanofibre adsorbents are a non-woven fibre matrix of high surface area and porosity previously investigated as a bioseparation medium. The effects of compression and bed layers, and subsequent heat treatment after electrospinning cellulose acetate nanofibres were investigated using diethylaminoethyl (DEAE) or carboxylate (COO) functionalisations. Transbed pressures were measured and compared by compression load, COO adsorbents were 30%, 70% and 90% higher than DEAE for compressions 1, 5 and 10 MPa, respectively, which was attributed to the swelling effect of hydrophilic COO groups. Dynamic binding capacities (DBCs) at 10% breakthrough were measured between 2000 and 12,000 CV/h (2 s and 0.3 s residence times) under normal binding conditions, and DBCs increased with reactant concentration from 4 to 12 mg BSA/mL for DEAE and from 10 to 21 mg lysozyme/mL for COO adsorbents. Comparing capacities of compression loads applied after electrospinning showed that the lowest load tested, 1 MPa, yielded the highest DBCs for DEAE and COO adsorbents at 20 mg BSA/mL and 27 mg lysozyme/mL, respectively. At 1 MPa, DBCs were the highest for the lowest flowrate tested but stabilised for flowrates above 2000 CV/h. For compression loads of 5 MPa and 10 MPa, adsorbents recorded lower DBCs than 1 MPa as a result of nanofibre packing and reduced surface area. Increasing the number of bed layers from 4 to 12 showed decreasing DBCs for both adsorbents. Tensile strengths were recorded to indicate the mechanical robustness of the adsorbent and be related to packing the nanofibre adsorbents in large scale configurations such as pleated cartridges. Compared with an uncompressed adsorbent, compressions of 1, 5 and 10 MPa showed increases of 30%, 110% and 110%, respectively, for both functionalisations. The data presented show that capacity and mechanical strength can be balanced through compression after electrospinning and is particular to different functionalisations. This trade-off is critical to the development of nanofibre adsorbents into different packing configurations for application and scale up in bioseparation.

## Highlights

• Optimised chemistry protocols of DEAE and COO functionalisations.
• Compression applied after electrospinning improved mechanical strength.
• Increasing compression and bed layers lowered binding capacities.

## 1 Introduction

The contribution of biotechnology products to the global prescription and over-the-counter pharmaceutical markets were estimated to be worth $118 billion in 2011 with increased focus in the therapy areas of oncology, anti-diabetes and vaccines [1]. Some individual products are reaching annual sales of over $1 billion [2]. As the market moves towards developing more complex biomolecules such as fusion proteins and antibody fragments, purification stages in downstream processing are becoming more expensive. The advancement of cell line engineering in upstream processing, including transfection methods and media development, in upstream processing have realised increased product titres over the past two decades [3]. However, downstream processing has yet to achieve a dramatic improvement in process efficiency partly due to limitations in widely used packed-bed resins including diffusive mass transfer, achievable flow rates and scale-up volumes. Protein bioseparation media using convective mass transfer such as porous membranes and monoliths have received increased attention because they avoid this diffusion limitation and have a higher capture efficiency and reduced buffer use to improve overall productivity [4]. In the last 30 years, rigid porous monoliths have also been introduced and developed. The single solid continuous matrix has no interstitial voids and can also vastly improve productivity by operating at much higher flowrates than packed-bed chromatography [5]. Current advantages in industry have been realised in the polishing stage of monoclonal antibody purification using flowthrough mode where a membrane column binds impurities and allows the target to pass through [6].

**Fig. 1:**
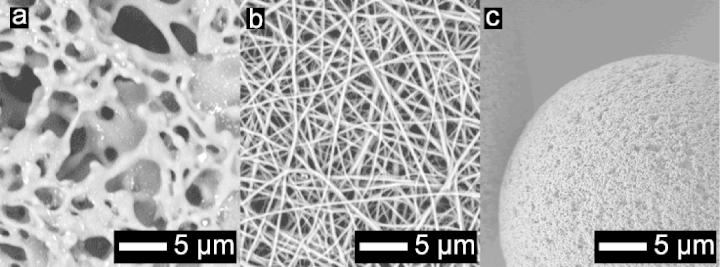
Scanning electron microscopy images comparing protein purification media. (a) Sartobind S cellulose membrane (Sartorius Stedim, Epsom, UK). (b) Compressed and heat treated regenerated cellulose nanofibre adsorbent. (c) Fractogel EMD TMAE HiCap packed-bed resin (EMD Millipore, Darmstadt, Germany) with 40–90 μm bead diameters and approximately 0.1 μm pore diameter.

Nanofibre electrospinning involves passing a viscous polymer solution through a microneedle charged at a high voltage (>5 kV) to deposit a continuous fibre strand to a grounded collector and form a non-woven mat with a fibre diameter of less than 1 μm [7]. Electrospun nanofibres have been investigated for a multitude of applications including tissue engineering [8], catalysis and sensors [9], [10], filtration [11] and composites [12]. Cellulose is a commonly used material in membrane chromatography and filtration for being chemically resistant, cheap and has good non-specific binding properties [4]. However, cellulose raises many challenges in electrospinning because it is difficult to dissolve and the solvent systems required can lead to non-uniform nanofibre deposition [13]. As such, electrospinning readily dissolvable cellulose derivatives such as cellulose acetate are preferred followed by regeneration to cellulose via hydroxide treatment. For uniform fibre deposition of cellulose acetate, controlling polymer solution (viscosity), flow rate and voltage as well as environmental conditions have been shown to be critical [14]. Annealing cellulose acetate nanofibres with heat is a common step to improve mechanical strength by creating “spot welds” at fibre strand overlap points. Fig. 1 shows scanning electron microscopy (SEM) images of the different morphologies for a cast porous membrane, packed-bed resin and an annealed electrospun regenerated cellulose nanofibre adsorbent. A nanofibre adsorbent balances a high surface area and porosity with the benefits of convective mass transfer.

Chemical modifications of chromatographic media using hydroxyl groups on the support for application in bioseparation have been researched [15]. Electrospun nanofibre adsorbents in bioseparation have been reported for cellulose [16] and other polymers including polysulfone [17] and polyacrylonitrile [18]. Diethylaminoethyl (DEAE) cellulose electrospun nanofibres have been fabricated by Williamson ether synthesis using 2-(diethylamino) ethyl chloride hydrochloride (DAECH) to show improved separation productivity compared with porous membranes [19], [20]. Alcohol groups on cellulose have been controllably oxidised to carboxylate (COO) groups using (2,2,6,6-tetramethylpiperidin-1-yl)oxyl (TEMPO) as a catalyst and sodium hypochlorite as the oxidant [21], [22]. The main application of TEMPO-mediated oxidation is in the preparation of nanocellulose from wood pulp where the ionic repulsion of COO groups helps force cellulose fibres apart during processing, reducing the mechanical energy required [23]. The use of TEMPO-mediated oxidised electrospun cellulose nanofibre has been used before to bind metal ions [22] and viruses [24]. The physical and chemical methods applied in fabricating electrospun cellulose nanofibre adsorbents affect bioseparation performance and controlling parameters is important to fabricating a reproducible material. Compressing nanofibre sheets combined with annealing via heat treatment is used to further improve mechanical properties than heat treatment alone. A robust nanofibre adsorbent is essential in scaled up packed bed configurations such as pleated cartridges, as seen in membrane chromatography. However, chemical modifications applied to nanofibres may adversely affect morphology and structure.

## 2 Materials and methods

### 2.1 Fabricating cellulose nanofibre adsorbents

To shorten the electrospinning time and produce nanofibre mat of consistent bed height, four microneedles (100 mm length; 0.5 mm i.d.) were used and the collector was moved side-to-side in line with the needle array. The operating voltage was 30 kV, the humidity set to 70% and temperature to 25 °C. A 20 wt.% cellulose acetate (Mr = 29,000, 40% acetyl groups, Sigma-Aldrich, Dorset, UK) solution was prepared in acetone:DMF:ethanol (Sigma–Aldrich) at a ratio of 2:2:1 as previously described [14], [19]. The solution was spun at 2.5 mL/h for 10 h. The collector was a rotating drum (200 mm dia.; 300 mm length) set at 60 rpm on a translation stage set at 300 mm *x*-axis displacement (150 mm either side of the needle array centre) at a rate of five loops per minute. A sheet 600 mm × 180 mm was produced equating to approximately 30 g/m^2^, which was comparable to the nanofibre mat used in our previous study but a reduction in spinning time from 36 h to 10 h [19]. Squares (80 mm × 80 mm) were cut, layered and placed in between two 100 mm × 100 mm square aluminium blocks to act as the die. Compression was performed for 2 min in a manual hydraulic press (Specac, Kent, UK) under different loads of 1000, 5000 and 10,000 kg as indicated on the gauge, corresponding to 0.98, 4.9 and 9.8 MPa, respectively, for brevity, we used rounded up values; 1, 5 and 10 MPa, respectively. To study the effects of the main reactant concentration, 8 layers were compressed at a load of 5 MPa. To study the effects of physical modification, 8 layers were compressed at loads of 1, 5 and 10 MPa and 4, 8 and 12 layers were compressed at 5 MPa. The nanofibre sheet was immediately placed in a preheated oven (NR30F, Carbolite, Sheffield, UK) set at 213 °C for 30 min. Cellulose acetate mats were regenerated to cellulose by deacetylation using 0.1 M sodium hydroxide in 2:1 H_2_O:EtOH overnight. The addition of ethanol was essential to ensure complete deacetylation.

### 2.2 DEAE modification

Cellulose adsorbents can be reacted directly with DAECH to form DEAE ligands via alkylation [19], [20]. A reaction solution of 100 mL deionised water was employed with varying concentrations of DAECH at 50 and 200 mmol/g cellulose stirred at 250 rpm to avoid damaging the nanofibre mat. To improve DEAE functionalisation, a repeat reaction was performed using 200 mmol DAECH per gram cellulose (2× 200 mmol) as previously reported [25]. The reaction was stirred for 15 min at 250 rpm. Then the mat was treated in hot (90 °C) 0.5 M NaOH solution for 10 min to complete the reaction and dissolve any unwanted reactants. The DEAE-cellulose adsorbent was rinsed in copious amounts of water.

### 2.3 TEMPO-mediated oxidation

A COO-cellulose adsorbent was produced following a procedure modified from that previously reported [21]. A 100 mL aqueous mixture of TEMPO (0.002 g; Sigma-Aldrich) and NaBr (0.02 g; Sigma-Aldrich) was adjusted to a pH of 10.5 using aqueous 0.1 M NaOH. The nanofibre mat was stirred for 5 min. A syringe pump was used to dropwise add sodium hypochlorite (NaClO; Sigma-Aldrich) for the three concentrations investigated; 5, 10 and 20 mmol sodium hypochlorite (NaClO) per gram cellulose. The pH was monitored to ensure pH remained above 10.5 to encourage oxidation of the C6 hydroxyl on the cellulose. The time taken to add NaClO was 10 min and the mixture was allowed to stir for a further 5 min. Ethanol (10 mL, Sigma-Aldrich) was added to quench the reaction and stirred for 10 min. The mat was washed thoroughly with ultrapure water. To oxidise any remaining aldehyde groups, sodium chlorite (Sigma-Aldrich) treatment (0.45 g in 45 mL in 1 M acetic acid) was performed for 48 h in the dark to as previously described [26].

### 2.4 Morphological, chemical and tensile strength analyses

SEM imaging was performed using a Phenom G2 Pro (Phenom-World BV, Eindhoven, The Netherlands) at an accelerating voltage of 10 keV. Images were captured and analysed with Firbometric software (Phenom-World BV) to estimate fibre diameter. Fourier transform infra-red attenuated total reflectance (FTIR-ATR) was used to characterise the chemical group changes on a Thermo Scientific Nicolet iS10 FT-IR Spectrometer (Loughborough, UK). Spectra were recorded from dry samples in the range 4000–500 cm^−1^ by an accumulation of 50 scans. A background was measured with 10 scans prior to each sampling. For ultimate tensile strength measurements, compressed adsorbent samples, 1, 5 and 10 MPa, were cut into 15 mm × 10 mm (*L* × *W*) strips and placed into a tensometer. The strips were stretched at 1 mm/min and the highest recorded force before breaking was used.

### 2.5 Equilibrium adsorption capacities

To assess the nanofibre equilibrium binding capacity the mats were cut into 25-mm discs. Discs were incubated in 0.0–2.0 mg/mL model protein solutions and UV absorbance readings at 280 nm (Jasco V-630, Essex, UK) were taken at each step of before binding, after binding (16 h), wash (1 h) and elution (1 h). For testing the DEAE cellulose nanofibre, BSA was used as the model protein in 10 mM Tris buffer at pH 8.0. For the COO-cellulose binding study, lysozyme was used in 20 mM sodium acetate buffer at pH 5.5. The elution buffers used were the same as the respective binding buffer and containing 0.5 M NaCl. After elution, the nanofibres were regenerated in 0.1 M aqueous NaOH for repeat testing. Three tests were performed and the adsorbed equilibrium protein concentrations (*Q*) and liquid phase equilibrium concentrations (*C*) were averaged. The Langmuir adsorption isotherm *Q* = *Q*_max_*K*_*d*_*C*/*K*_*d*_ + *C* was used, where *Q*_max_ is the maximum capacity of protein bound, and *K*_*d*_ is the equilibrium dissociation constant. The linearised form of Langmuir isotherm was plotted and from a line of best fit the *Q*_max_ and *K*_*d*_ values were estimated. The wet bed height was measured with a digital micrometer (Mitutoyo, Kawasaki, Japan; 0.001 mm resolution) to calculate the volume. The experiment was performed three times. Elution performance was calculated as a ratio of the protein concentration after elution and the adsorbed equilibrium protein concentration. The recovered protein concentration was 75% for DEAE and 90% for COO adsorbents.

### 2.6 DBCs and transbed pressures

The dynamic binding capacity (DBC) was measured using an AKTA Basic (GE Healthcare, Uppsala, Sweden) system with UV measurement at 280 nm. A custom-made 25-mm PEEK filter holder was previously designed using frit spacers to ensure full radial flow distribution across an adsorbent at very high flowrates [19]. The buffers and model proteins were the same as used in the equilibrium adsorption study. The nanofibre adsorbent in the filter holder was well equilibrated prior to binding. The binding flowrates were varied between 10 and 610 cm/h. BSA or lysozyme at a concentration of 2 mg/mL in a 2-mL sample loop was injected for DEAE and COO adsorbents testing in most cases. For 8-layer adsorbents compressed at 1 MPa all of the protein injected was bound to the adsorbent and the protein concentration was increased to 3 mg/mL and 5 mg/mL of BSA and lysozyme to provide a maximum DBC for the DEAE and COO adsorbents, respectively. Elution was performed with a 30% mix of 1 M NaCl in respective binding buffer at a 610 cm/h and the adsorbent was further cleaned with elution buffer at 610 cm/h, followed by re-equilibration with binding buffer. The DBC was calculated at 10% breakthrough using the following equation DBC10% = ((*V*_10%_ − *V*_0_) + *C*_Load_)/*V*_Bed_ where *V*_0_ is the void volume of the entire system, *C*_Load_ is the concentration of the protein solution loaded, and *V*_10%_ is the volume of sample that must be loaded before achieving 10% breakthrough. *V*_Bed_ is the bed volume in millilitre measured from a bed height range between 0.014 cm and 0.07 cm with a digital micrometer (Mitutoyo). Bed volumes were taken as an average of the three adsorbents tested. For DEAE adsorbents: 1 MPa, 0.35 mL; 5 MPa (8 layers), 0.16 mL; 10 MPa, 0.16 mL; 4 layers, 0.07 mL; and 12 layers, 0.21 mL. For COO adsorbents: 1 MPa, 0.34 mL; 5 MPa (8 layers), 0.19 mL; 10 MPa, 0.20 mL, 4 layers, 0.09 mL and 12 layers, 0.21 mL. Blank tests using non-pressed unmodified cellulose adsorbents were performed as controls. To measure transbed pressure, the AKTA was programmed to increase in flowrate in steps up to 50 mL/min and presented as the recorded back pressure minus the system back pressure including a filter holder containing no adsorbent.

## 3 Results and discussion

### 3.1 Surface morphology

**Fig. 2:**
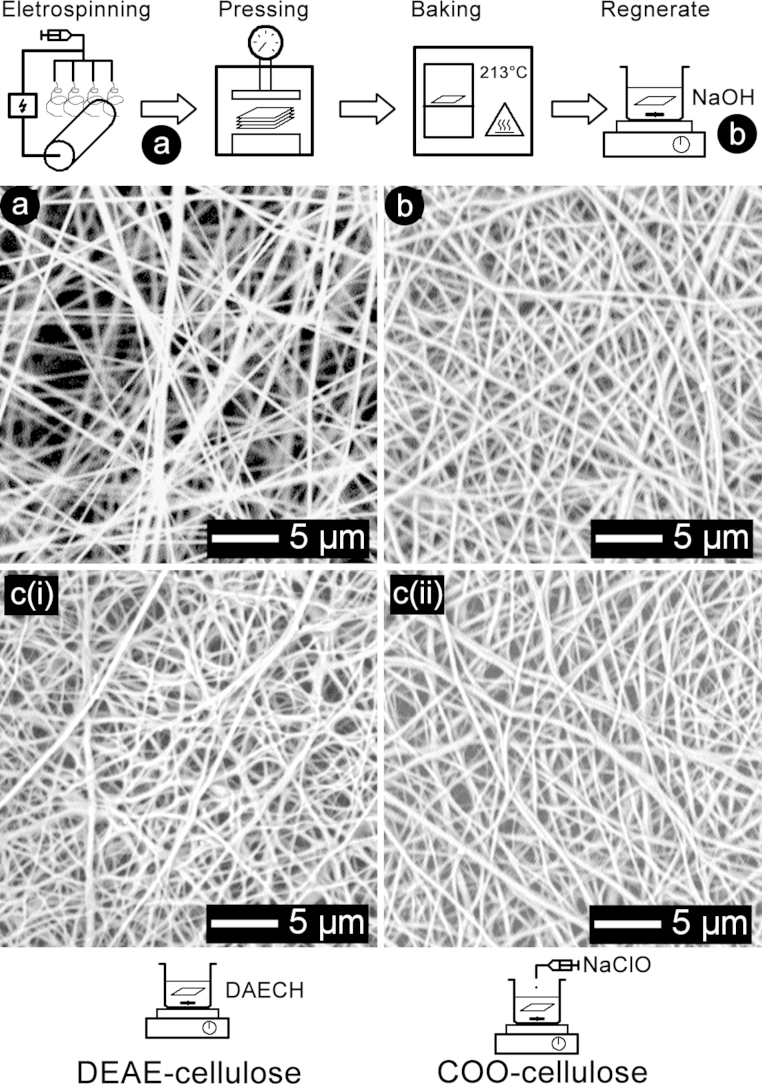
Fabrication of electrospun cellulose nanofibre adsorbents and representative scanning electron microscopy images. (a) Cellulose acetate nanofibre mat before any modification. (b) Regenerated cellulose adsorbent after 5 MPa compression, heat treatment and deacetylation. (c)(i) Diethylaminoethyl (DEAE) cellulose adsorbent. (c)(ii) Carboxylate (COO) cellulose adsorbent.

SEM serves as an important tool for investigating morphologies and fibre diameters of electrospun nanofibres. Fig. 2 shows representative SEM images and modification steps taken to fabricate DEAE and COO adsorbents. The cellulose acetate starting material prior to any compression or baking was fragile to handle and had a cotton-wool like texture. The morphology appeared open with large black spaces between the straight fibre strands (Fig. 2a). The electrospinning conditions were previously reported and the same small range in nanofibre diameters was measured here at an average of 0.5 μm [14]. Eight layers of cellulose acetate were compressed at 5 MPa, oven-baked and deacetylated to produce regenerated cellulose. The general appearance was a more compact matrix with strands retaining a linear appearance and diameters remained in the same range (Fig. 2b). Figs. 2c(i) and c(ii) show fibre matrices following chemical modifications to DEAE by repeat treatment of DAECH (2× 200 mmol) and COO groups at 20 mmol NaClO concentration per gram adsorbent, respectively. DEAE adsorbents showed a slightly distorted appearance with fibre strands losing some of their linear character, which has not been reported for singly treated DEAE nanofibres in other studies [19], [20]. More than two repeats of DAECH treatment using the above protocol led to a complete degradation of the nanofibre matrix upon drying, becoming a hard opaque material. COO adsorbents also show a loss of linear character and distortion not seen in the unmodified regenerated cellulose matrix. TEMPO-mediated oxidation has use in the nanofibrillation of wood pulp to prepare nanocellulose particles and fibrils. By charging the cellulose polymer chains with COO groups the process of homogenising wood pulp consumes less energy because ionic repulsion forces assists in fibril separation [23]. The ionic repulsion is used to degrade wood fibrils into nanofibrils 3–4 nm in diameter [27]. In the DEAE and COO nanofibre adsorbents here, the effect of charged groups on the nanofibre strand in solution may lead to ionic repulsion forcing nanofirils apart that make up the strand becoming noticeable. Upon drying for SEM analysis the appearance of the nanofibre matrix was only slightly distorted because the surface was no longer charged. The chemical conditions of 2× 200 mmol DAECH for DEAE and 20 mmol NaClO for COO adsorbents were considered the highest possible before any considerable change in the morphology rendered the adsorbent unsuitable for application.

### 3.2 FTIR-ATR

**Fig. 3:**
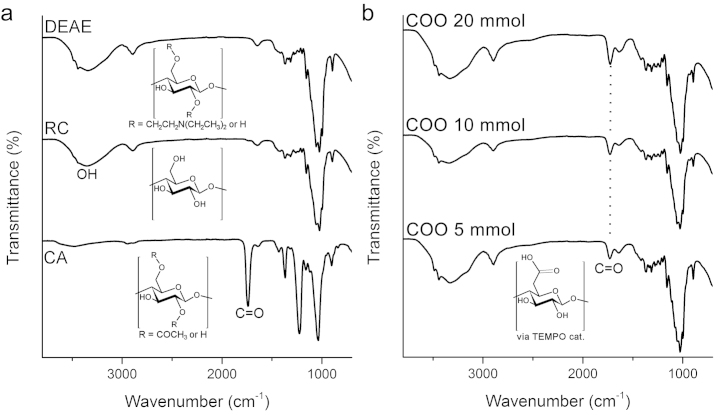
FTIR-ATR spectra of diethylaminoethyl (DEAE) and carboxylate (COO) cellulose adsorbents showing the change in chemical groups during adsorbent fabrication. (a) Cellulose acetate (CA) starting material, regenerated cellulose (RC) and DEAE cellulose adsorbent. (b) COO modification shows an increasing height of the carbonyl peak (C=O) from carboxylate salt group (COONa) with increasing concentrations of NaClO applied.

FTIR-ATR was employed to investigate the changes in chemical groups on the surface of cellulose nanofibre adsorbents throughout the different modification and representative spectra are shown in Fig. 3. Cellulose acetate deacetylation to regenerated cellulose was clear from the replacement of acetate peaks (ester carbonyl (C=O); 1740 cm^−1^, carbon-methyl (CCH_3_); 1365 cm^−1^ and ester linkage (OC=O); 1221 cm^−1^) by a broad and larger alcohol (OH) peak in the 3300–3500 cm^−1^ region. DEAE modification (2× 200 mmol) showed no new peaks because the weak stretching and relatively low concentration of the tertiary amine bonds were masked by the cellulose peaks, regardless how high a modification is used [19]. Fig. 3b shows that COO-cellulose created a new peak at 1731 cm^−1^, corresponding to the carbonyl group of the carboxylate salt (COONa). As the amount of oxidant NaClO was increased, the C=O peak height increased, indicating an increase in COO groups. The application of FTIR-ATR was convenient to investigate groups with detectable peaks.

### 3.3 Tensile strength

**Fig. 4:**
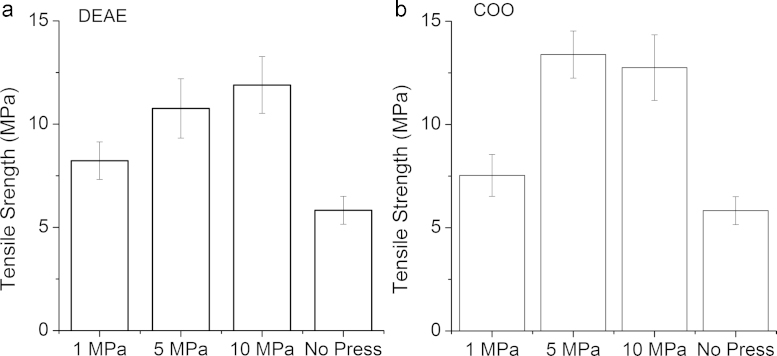
Tensile strengths of diethylaminoethyl (DEAE) and carboxylate (COO) cellulose adsorbents increased with increasing compressions applied during fabrication. The ‘No Press’ shown was eight layers of uncompressed regenerated cellulose. Error bars indicate ±SD.

Previous investigations into non-compressed nanofibre adsorbents suffered damage during chemical modification from mixing and thus required a less vigorous approach, hindering the amount and homogeneity of functionalisation. Compressing after electrospinning and followed by heat treatment was therefore found to be useful to reduce failures and produce consistent adsorbents. Samples were compared with non-compressed and heat treated regenerated cellulose indicated as the ‘No Press’ sample (Fig. 4). DEAE and COO adsorbents compressed at 1 MPa showed a 40% and 30% increase in tensile strengths compared with the non-compressed sample, respectively. Increasing compression to 5 and 10 MPa improved tensile strength over non-compressed further, recording percentage differences of 85% and 105% for DEAE and 130% and 120% for COO, respectively. However, the large standard deviation errors for 5 and 10 MPa compressions suggest no statistical difference between them and may indicate a maximum of tensile strength achieved for these cellulose adsorbents. The samples were tested when dry and the weak ion-exchange groups of DEAE and COO would be in their neutral form and not exhibiting any pronounced effect from ionic repulsion. TEMPO-mediated oxidation has been used in nanocellulose production, assisting fibre degradation during mechanical processing in solution through ionic repulsion [27]. The changes in morphology between chemical modifications were negligible and similarities in tensile strengths implied that chemical modification has little or no effect, at least in this sample size. Improving the mechanical strength of adsorbents contributes to creating a robust material capable of being handled and packed into large scale pleated or spiral-wound configurations.

### 3.4 Transbed pressures of varying bed layers and compressions

**Fig. 5:**
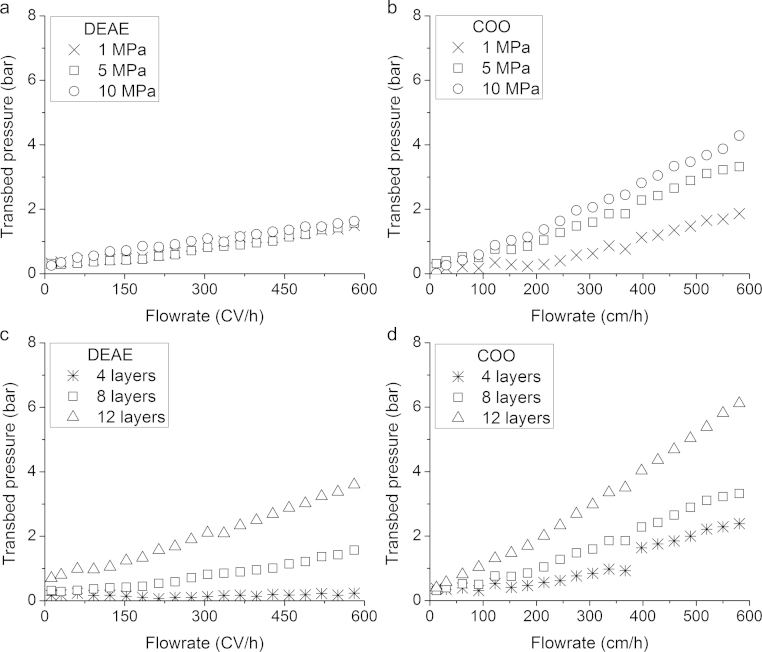
Transbed pressures of electrospun diethylaminoethyl (DEAE) and carboxylate (COO) cellulose nanofibre adsorbents of varying bed layers and compressions. (a) and (b) Eight-layer DEAE and COO adsorbents at varying compressions. (c) and (d) DEAE and COO adsorbents of varying bed layers compressed at 5 MPa.

Transbed pressures were recorded for increasing flowrates using adsorbents prepared at chemical modifications of 2× 200 mmol DAECH for DEAE and 20 mmol NaClO for COO adsorbents. Fig. 5a shows similarly increasing transbed pressures for varying compressions of 8-layer DEAE adsorbents with increasing flowrate with 10 MPa showing the highest of the three. Increasing compression during fabrication was expected to increase transbed pressures because the nanofibre matrix was more packed and porosity was reduced. DEAE transbed pressures were higher than non-compressed DEAE adsorbents previously reported [19]. COO adsorbents show considerably higher transbed pressures than DEAE and differences between increasing compression loads (Fig. 5b). COO groups are hydrophilic and have been previously shown to cause a higher back pressure when comparing carboxymethyl - with DEAE modified cellulose beads, suggesting this swelling effect contributes to increases in transbed pressure [28]. The change in matrix packing was clear in COO adsorbents emphasised by the hydrophilic nature of COO groups.

Porous membranes, like nanofibre adsorbents, are produced as flat sheets and designing a media with multiple layers to increase bed height is one method to increase bed volume. Transbed pressures of 4-, 8- and 12-layer adsorbents compressed at 5 MPa recorded noticeable differences with COO adsorbents showing twice as high pressures than DEAE (Fig. 5c and d). The 12-layer adsorbent bed volume was similar to 8-layer, but the packing of nanofiber matrix much higher, which was evidenced in the increase of transbed pressure. The 4-layer adsorbents were of such small bed volumes that hardly any pressure was recorded. Transbed pressures provided an insight to the packing of nanofibres, which had clearly increased with increasing compression from the tensile strength results. However, higher transbed pressures may reduce capacity because of the channelling effect seen in porous membranes where the proteins are not accessing all the surface area of an adsorbent.

### 3.5 Equilibrium absorption capacities of varying chemical modifications

**Fig. 6:**
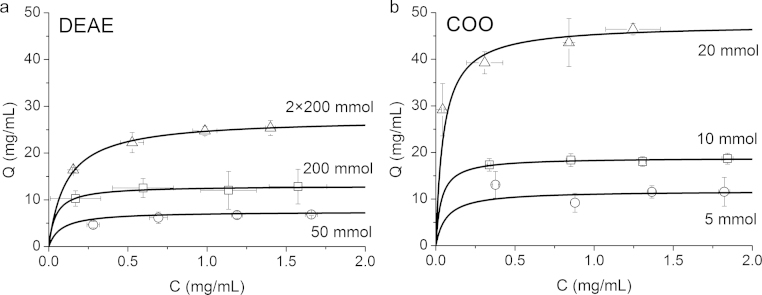
Equilibrium binding adsorption isotherms of 8-layer electrospun diethylaminoethyl (DEAE) and carboxylate (COO) cellulose nanofibre adsorbents compressed at 5 MPa and modified under different reactant concentrations. (a) BSA in 10 mM Tris buffer at pH 8 was used for DEAE testing and (b) lysozyme in 20 mM acetate buffer at pH 5.3 was used for COO. Error bars indicate ±SD of the average *Q* and *C* values taken from three replicates.

Reactant concentrations were varied to investigate capacities using 8-layer adsorbents compressed at 5 MPa (Fig. 6 and Table 1). DEAE modifications were varied with DAECH concentrations of 50 and 200 mmol/g cellulose and a repeated treatment (2× 200 mmol). The *Q*_max_ and *K*_*d*_ values were evaluated using the linearised form of Langmuir isotherm. However, lacking data in the low concentration region of the isotherm detracted from the reliability of the *K*_*d*_ values. Capacity was increased from the 50 mmol to 200 mmol adsorbent, showing that functionalisation can be controlled using DAECH amount. The 200 mmol concentration *Q*_max_ of 13 mg BSA/mL was similar to that we previously recorded for an uncompressed nanofibre adsorbent [19] but was lower than that of Zhang et al. [20]. Repeating the DAECH treatment has been previously shown to increase adsorbent capacity and the equilibrium capacity here was increased to 27.4 mg BSA/mL, which was twice that of single treatment [25]. COO nanofibre adsorbents showed an increase in *Q*_max_ for increasing concentrations of oxidising reagent, NaClO (Fig. 6b and Table 1) and agrees with the increased number of COO groups suggested in the FTIR spectra (Fig. 3b). The 20 mmol *Q*_max_ of 47.5 mg lysozyme/mL was comparable to some previously reported values for commercially available packed-bed resins, which typically have exceptionally high surface areas [29]. The swelling effect noted in the transbed pressure tests was difficult to account for under equilibrium binding conditions and may contribute to increasing capacity. Other COO nanofibre adsorbents have been studied for electrospun polyacrylonitrile where Chiu et al. achieved an equilibrium binding capacity using lysozyme of a similarly high value as in this study [30]. Polymer grafting techniques have shown advantages in vastly improving protein binding capacities [31]. An equilibrium capacity range using a nanofibre adsorbent was shown to be 4–25 times higher than that in this study, also using lysozyme and dependent on the amount of polymer grafted [16]. These studies reporting high capacity values support using the high surface of nanofibres as an adsorbent medium.

**Table 1:** Equilibrium binding study of the varying chemical modifications used to fabricate diethylaminoethyl (DEAE) and carboxylate (COO) cellulose nanofibre adsorbents, detailing the maximum capacity of protein bound, *Q*_max_, and dissociation constant, *K*_*d*_, estimated using a Langmuir linear regression fit.

Sample	DEAE cellulose			COO cellulose		
Reactant conc.	50 mmol	200 mmol	2× 200 mmol	5 mmol	10 mmol	20 mmol
*Q*_max_ (mg/mL)	7.5	13.0	27.4	11.8	18.9	47.5
*K*_*d*_ (mg/mL)	0.077	0.044	0.11	0.065	0.034	0.049

### 3.6 DBCs of varying chemical modifications

**Fig. 7:**
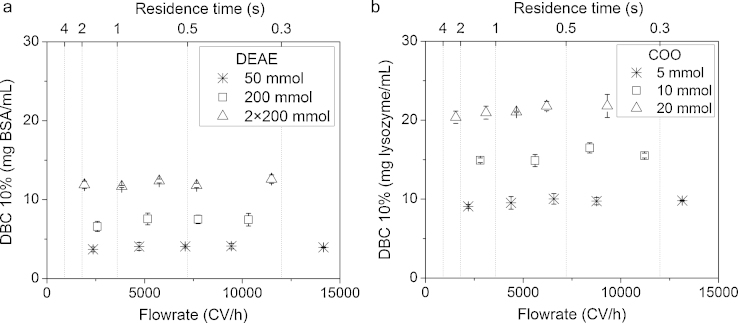
Dynamic binding capacities at 10% breakthrough of 8-layer electrospun (a) diethylaminoethyl (DEAE) and (b) carboxylate (COO) cellulose nanofibre adsorbents compressed at 5 MPa and modified under different reactant concentrations using identical binding conditions as before with NaCl elution. Error bars indicate ±SD.

An AKTA system and custom filter holder were used to estimate the DBCs at 10% breakthrough at varying flowrates of the same adsorbents studied above (Fig. 7). The residence times are shown to exemplify how little time is required for convective mass transfer of the target protein with a nanofibre adsorbent and ranged from 4 s to 0.3 s. The highest DBCs were recorded for the highest functionalisations found in the equilibrium binding study and were 12 mg BSA/mL for 2× 200 mmol DEAE and 21 mg lysozyme/mL for 20 mmol COO. At these DBCs, nanofibre adsorbents compare poorly against packed-bed media, which are typically in the 30–85 mg/mL range for DEAE and 40–100 mg/mL for COO and carboxymethyl resins, despite suggesting reasonable surface area for binding in the equilibrium study [32]. There was a large capacity drop between the *Q*_max_ values and DBCs. DBCs plateaued for increasing flowrates, where in traditional packed-bed chromatography we would expect continual loss through flow distribution effects on the diffusion mass transfer. This difference has been previously reported for a non-pressed DEAE nanofibre adsorbent tested under similar conditions [19]. Flow distribution properties of the custom filter holder used were considered as an explanation for the difference between *Q*_max_ values and DBCs. The filter holder was developed using spacer frits for complete flow distribution across an adsorbent and tested visibly using coloured dyes. Such tests would not be able to reveal the internal microscale structure of nanofibre matrix and some areas may be unreachable for binding under flow conditions but available under static conditions. Another contributing factor would be the chemical nature of the ion-exchange groups. The hydrophilic nature of the COO group and the swelling effect attributed to causing higher transbed pressure may allow for greater capacity under static conditions if a gel-like layer was formed around nanofibre strands. However, this reasoning can only be applied to the COO adsorbents and not DEAE. Further investigation into the structure of ion-exchange nanofibre adsorbents is required. Convective mass transfer media and particularly the non-dead-end structure of nanofibre adsorbents, have the ability to operate at considerably higher flowrates which benefits the overall productivity to separate proteins [19], [33]. Therefore a high dynamic capacity was less important than attaining a repeatable capacity at higher flowrates where in large scale devices, capacity can be circumnavigated with higher volumes of adsorbent.

### 3.7 DBCs of varying bed layers and compressions

**Fig. 8:**
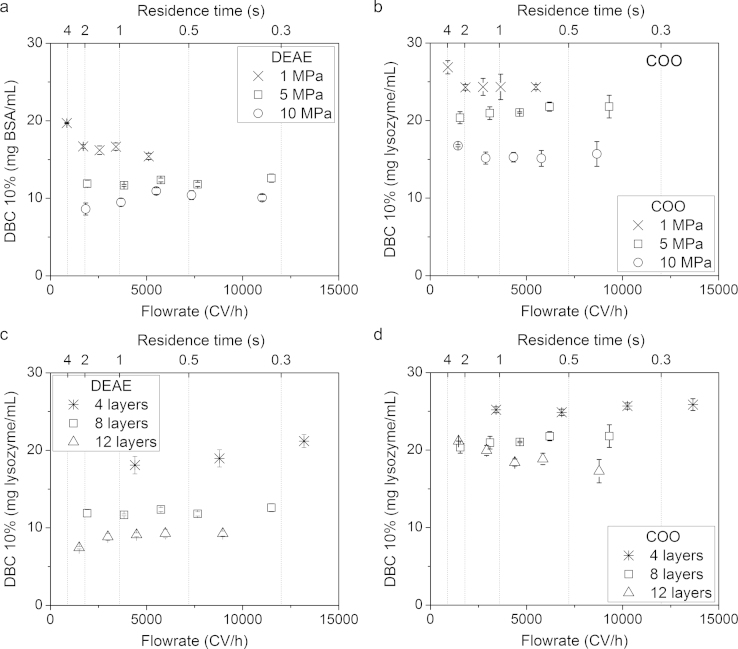
Dynamic binding capacities at 10% breakthrough of electrospun diethylaminoethyl (DEAE) and carboxylate (COO) cellulose nanofibre adsorbents for varying bed layers and compressions using identical binding conditions as before with NaCl elution. (a) and (b) Eight-layer DEAE and COO adsorbents at varying compressions. (c) and (d) DEAE and COO adsorbents of varying bed layers compressed at 5 MPa. Error bars indicate ±SD.

DBCs at 10% breakthrough for 8-layer adsorbents compressed at loads of 1, 5 and 10 MPa applied after electrospinning and adsorbents of varying bed layers (4, 8 and 12) compressed at 5 MPa were investigated for functionalisations 2× 200 mmol DEAE and 20 mmol COO (Fig. 8). The DEAE and COO chemistry protocols were different in capacities with adsorbents compressed at 1 MPa recording the highest DBCs at the lowest flowrate tested of 900 CV/h recording 20 mg BSA/mL and 27 mg lysozyme/mL, respectively. A decreasing capacity from 900 CV/h to around 2000 CV/h was present and then DBC appeared to stabilise for up to 12,000 CV/h. The flow distribution through the filter holder could be responsible for this initial capacity drop for increasing flowrate but DBCs were generally comparable for increasing flowrates over 2000 CV/h. The low transbed pressures of 1 MPa DEAE and COO suggested they create little resistance to flow and discourage channelling effects as a cause of the DBC drop. A detrimental effect of compression on DBC was found for increasing loads 5 MPa and 10 MPa with DBCs decreasing from 12 to 9 mg BSA/mL and 20 to 17 mg lysozyme/mL for DEAE and COO, respectively. The increasing loads would pack the adsorbents more and this reduced available surface area for protein binding would lead to lower DBCs. The DBCs were stable across all flowrates tested and combined with the improved mechanical strength properties, and then a loss in DBC could be considered an adequate trade-off for large scale application.

DEAE and COO adsorbents of varying bed layers compression at 5 MPa showed high DBCs at 10% breakthrough for the 4-layer adsorbents where the bed volume was very low at 0.07 mL and 0.09 mL, respectively. Increasing the number of bed layers for DEAE adsorbents showed reduced DBCs. For COO 12-layer adsorbents recorded similar DBCs to 8-layer up to 3000 CV/h but then DBCs began to decrease and may suggest that channelling effects were present due to the high transbed pressures. The bed layer results indicated a second consideration in fabricating nanofibre adsorbents where compression at 5 MPa can create a robust material but increasing the bed volume with layers further reduced DBCs.

## 4 Conclusions

Compression and heat treatment steps during the fabrication of nanofibre adsorbents allow their physical properties to be tuned towards their application as a chromatography medium. Mechanical properties are critical for handling and packing into large scale devices and impact operable flowrates, column capacity and hence batch operation time. Functionalisations also directly affect these bioseparation properties. The differences between DEAE and COO modifications are clear as shown by changes in morphologies, transbed pressures and capacities. Absorbent tensile strengths were similar for DEAE and COO and were found to increase with greater levels of compression after electrospinning with no significant difference between functionalisations. Transbed pressures show seemingly little effect between compressing loads of DEAE and yet large changes for COO, which is attributed to the hydrophilic COO groups. When studying protein separation the highest attainable capacities by functionalisation were found as a repeated treatment of 200 mmol/g adsorbent DAECH for DEAE and 20 mmol/g NaClO for COO adsorbents. Nanofibres prepared at the lowest level of compression (1 MPa) yielded the highest DBCs at the lowest flowrate, which indicates the available surface area for binding. At 5 and 10 MPa compressions capacity was decreased and increasing bed layers compressed at 5 MPa also decreased DBCs. However, DBCs recorded remained stable for increasing flowrate at 5 and 10 MPa compressions while 1 MPa was only stable above 2000 CV/h.

This study shows that the interactions between fabrication and functionalisation in the synthesis of nanofibre adsorbents are critical to the required physical properties of the material for packing and operating a bioseparation medium. This requires that nanofibre materials properties are measured and understood alongside developments in surface chemistry, in order to strike the correct balance of capacity and material strength and tailor the material to the application.
